# Syphilis Infections Among Women of Reproductive Age in the Southern United States: An Analysis From the Study of Treatment and Reproductive Outcomes (STAR)

**DOI:** 10.1093/ofid/ofag190

**Published:** 2026-04-06

**Authors:** Madison Sophia Meyer, Erin Rebecca Carr, Candice A Sternberg, Paola B Fernandez, Laura Beauchamps, Patricia Raccamarich, JoNell Potter, Daniel Westreich, Seble Kassaye, Elizabeth F Topper, Aadia Rana, Deborah Konkle-Parker, Deborah L Jones, Anandi N Sheth, Maria L Alcaide

**Affiliations:** Division of Infectious Diseases, Department of Medicine, University of Miami Miller School of Medicine, Miami, Florida, USA; Department of Medicine, School of Medicine, Wayne State University, Detroit, Michigan, USA; Division of Infectious Diseases, Department of Medicine, University of Miami Miller School of Medicine, Miami, Florida, USA; Department of Medicine, Brigham and Women's Hospital, Boston, Massachusetts, USA; Division of Infectious Diseases, Department of Medicine, University of Miami Miller School of Medicine, Miami, Florida, USA; Division of Infectious Diseases, Department of Medicine, University of Miami Miller School of Medicine, Miami, Florida, USA; Division of Infectious Diseases, Department of Medicine, University of Miami Miller School of Medicine, Miami, Florida, USA; Division of Infectious Diseases, Department of Medicine, University of Miami Miller School of Medicine, Miami, Florida, USA; Department of Obstetrics, Gynecology and Reproductive Sciences, University of Miami Miller School of Medicine, University of Miami, Miami, Florida, USA; Department of Epidemiology, University of North Carolina–Chapel Hill, Chapel Hill, North Carolina, USA; Department of Medicine, Georgetown University, Washington, DC, USA; Department of Epidemiology, Johns Hopkins Bloomberg School of Public Health, Baltimore, Maryland, USA; Division of Infectious Diseases, Department of Medicine, University of Alabama–Birmingham, Birmingham, Alabama, USA; Schools of Nursing, Medicine, and Population Health Sciences, University of Mississippi Medical Center, Jackson, Mississippi, USA; Department of Psychiatry and Behavioral Sciences, University of Miami Miller School of Medicine, Miami, Florida, USA; Division of Infectious Diseases, Department of Medicine, School of Medicine, Emory University, Atlanta, Georgia, USA; Division of Infectious Diseases, Department of Medicine, University of Miami Miller School of Medicine, Miami, Florida, USA

**Keywords:** HIV, reproductive-age women, social determinants of health, syphilis, vulnerable population

## Abstract

Syphilis is resurging among reproductive-age women in the United States. Among 773 women with and without HIV enrolled in the Study of Treatment and Reproductive Outcomes (STAR), 2.9% had prior/current syphilis, including 1 pregnant participant. Factors associated with prior/current syphilis included lower education, unemployment, incarceration history, and history of transactional sex.

Syphilis has resurfaced as a significant public health threat in the United States [1]. Despite available screening and treatment, syphilis cases have steadily increased since 2001 among men, women, and infants, creating a modern epidemic [2]. From 2013 to 2022, syphilis rates among reproductive-age women and congenital syphilis (CS) surged by 800% [3], and the average annual case rates increased from 12.5 to 78.0 per 100 000 among reproductive-age females [3].

Pregnant women with syphilis face heightened risks of preterm birth, emphasizing the critical need for enhanced public health interventions to safeguard maternal health and combat CS [4]. The rise in CS among racial and ethnic minorities directly reflects the disparities in syphilis cases among women of childbearing age, which persists despite available antenatal screening and treatment [1, 3]. In 2021, infants born to Black, Hispanic, or American Indian/Alaska Native mothers were 8 times more likely to have CS as compared with those born to White mothers [4].

In addition to the maternal and neonatal mortality associated with syphilis, syphilis increases the risk of HIV acquisition, which highlights the potential impact to communities at risk in the US South [5]. Regional and racial disparities in syphilis and HIV rates in women living in the United States arise primarily from social determinants of health, including education, income, stigma, and socioeconomic challenges, which are prevalent among reproductive-age women living in the US South and result in high-risk behaviors [6].

This study examines syphilis prevalence among women in the Study of Treatment and Reproductive Outcomes (STAR), the largest cohort of reproductive-age women with and without HIV (WWH and WWOH with risk factors for HIV acquisition) in the United States.

## METHODS

### Study Overview and Design

STAR is a longitudinal observational cohort of women aged 18 to 45 years, including WWH and WWOH. This report represents a cross-sectional analysis of baseline enrollment data. The University of Miami single Institutional Review Board (20190953) approved this study. Signed informed consent was obtained from all participants.

### Participants

Recruitment occurred at 6 sites in the US South (Atlanta, GA; Birmingham, AL; Chapel Hill, NC; Jackson, MS; Miami, FL; Washington, DC), as previously described [7].

### Measures

Data collection included interviewer-administered questionnaires (demographics, medical history, reproductive health, risk behaviors, and syphilis history), as well as blood and urine sample collection. Blood was tested for HIV and syphilis. Urine was tested for pregnancy and chlamydia and gonorrhea by nucleic acid amplification testing. Syphilis tests were done in commercial laboratories.

### Syphilis Testing

Syphilis testing included an initial nontreponemal test followed by confirmation with treponemal testing. Prior/current syphilis was determined when there was a reactive nontreponemal test followed by a reactive treponemal test.

The rapid plasma reagin (RPR) test was used as the nontreponemal serologic test, and when reactive, titration was performed [8]. The fluorescent treponemal antibody-absorption test, microhemagglutination assay for *Treponema pallidum* antibodies, and *Treponema*-specific IgG test were used as confirmatory treponemal tests [8].

As no physical examination was performed as part of the study and the study team did not have access to prior syphilis test results, syphilis history was determined through participant self-report and review of available medical records. Data on prior pregnancies were limited. Syphilis screening results and the timing of prior pregnancies relative to syphilis diagnosis were unavailable for this analysis. In the absence of physical examination findings and prior documented serologic titers, syphilis status was categorized by RPR criteria and participant-reported history. Higher nontreponemal titers (≥1:8) are more likely associated with active infection [8]. Prior/current syphilis was categorized by RPR criteria as follows: recent infection (no history of syphilis and RPR ≥1:8), recent reinfection/reactivation (history of syphilis with RPR ≥1:8), late latent syphilis (no history of syphilis and RPR <1:8), or serofast status (history of syphilis with RPR <1:8). RPR titers independently cannot distinguish reinfection from previously treated infection without prior longitudinal serologic data.

### Data Analysis

A cross-sectional analysis of baseline enrolled participants was performed. Descriptive statistics summarized participant characteristics and syphilis outcomes. Wilcoxon rank sum tests for continuous variables and χ^2^ or Fisher exact test for categorical variables were used for group comparisons between individual- and community-level factors and syphilis status. Statistical significance was set at an α level of .05, with analysis conducted with R version 4.5.1.

## RESULTS

Results are illustrated in Table 1. Participants were 773 reproductive-age women with a mean age of 34 years: 450 WWH and 323 WWOH. Most participants self-identified as non-Hispanic Black (74%), followed by Hispanic (11%), non-Hispanic White (10%), or other (4.4%). Over half of the participants (54%) were never married. Almost half of the participants (48%) were unemployed, with 40% reporting their monthly income as <$1500.

**Table 1.** Sociodemographic, Risk Behavior, Reproductive, and Sexually Transmitted Infections History Among STAR Participants With and Without Syphilis

	Participants, No. (%)			
Variable	Prior/Current Syphilis (n = 23)	No Syphilis (n = 750)	Total (N = 773)	*P* Value^a^
Age, y, mean (SD)	36 (6)	34 (7)	34 (7)	.344
Race and ethnicity				.345
Hispanic	5 (22)	82 (11)	87 (11)	
Non-Hispanic Black	17 (74)	553 (74)	570 (74)	
Non-Hispanic White	1 (4.3)	78 (10)	79 (10)	
Non-Hispanic other	0 (0)	34 (4.6)	34 (4.4)	
Educational attainment				**<.001**
Less than high school	6 (26)	143 (19)	149 (19)	
High school graduate or GED	15 (65)	257 (34)	272 (35)	
Some college or higher	2 (8.7)	349 (47)	351 (45)	
Marital status				.608
Married	2 (8.7)	116 (15)	118 (15)	
Not married but living with a partner	4 (17)	94 (13)	98 (13)	
Divorced/widowed/separated	3 (13)	122 (16)	125 (16)	
Never married	13 (57)	402 (54)	415 (54)	
Other	1 (4.3)	15 (2.0)	16 (2.1)	
Employment status				.055
Employed	7 (30)	396 (53)	403 (52)	
Unemployed	16 (70)	352 (47)	368 (48)	
Average household monthly income, US $				.812
≤1500	10 (45)	283 (40)	293 (40)	
1501–3000	7 (32)	218 (31)	225 (31)	
≥3001	5 (23)	202 (29)	207 (29)	
Difficulty in paying for basic needs (eg, food, shelter, heat)				.241
Very hard	2 (8.7)	141 (19)	143 (19)	
Somewhat hard	13 (57)	300 (40)	313 (41)	
Not very hard	8 (35)	307 (41)	315 (41)	
Age at first sexual encounter, y, mean (SD)	15.1 (3.3)	15.2 (3.7)	15.2 (3.7)	.574
No. of lifetime male sexual partners, median [IQR]	15 [5–50]	10 [5–20]	10 [5–20]	.095
No. of male sexual partners in the past year				.948
One primary male partner	11 (50)	363 (52)	374 (52)	
More than 1 male partner	9 (41)	279 (40)	288 (40)	
No male partner	2 (9.1)	59 (8.4)	61 (8.4)	
History				
Sex for drugs, money, or shelter in the past 5 y	5 (22)	64 (8.5)	69 (8.9)	.**046**
Incarceration	13 (57)	190 (25)	203 (26)	.**002**
Lifetime history of pregnancy	19 (83)	470 (63)	489 (64)	.087
Pregnancy status				.312
Currently pregnant	4 (17)	82 (11)	86 (11)	
Not currently pregnant	19 (83)	665 (89)	684 (89)	
HIV serostatus				.703
Persons with HIV	12 (52)	438 (58)	450 (58)	
Persons without HIV	11 (48)	312 (42)	323 (42)	
Lifetime history				
Chlamydia	7 (30)	209 (28)	216 (28)	.998
Gonorrhea	8 (35)	152 (20)	160 (21)	.115
Trichomoniasis	6 (26)	194 (26)	200 (26)	>.999
Current chlamydia infection				.352
Yes	1 (4.3)	11 (1.5)	12 (1.6)	
No	22 (96)	723 (98)	745 (98)	
Not tested	0 (0)	2 (0.3)	2 (0.3)	
Current gonorrhea infection				>.999
Yes	0 (0)	21 (2.9)	21 (2.8)	
No	23 (100)	711 (97)	734 (97)	
Not tested	0 (0)	2 (0.3)	2 (0.3)	
Current trichomoniasis infection	4 (18)	81 (12)	85 (12)	.314

Data are presented as No. (%) unless noted otherwise. Bold indicates *P* < .05.

Abbreviation: STAR, Study of Treatment and Reproductive Outcomes.

^a^Wilcoxon rank sum test was used for continuous variables. For categorical variables, Pearson ^2^ test was used for expected cell counts ≥5; otherwise, Fisher exact test was used.

About one-quarter (26%) reported having been incarcerated at least once in their lives. About 11% were currently pregnant and 64% had previously been pregnant. Among the participants, 23 (2.9%) had prior/current syphilis and 750 (97%) did not have syphilis. Among the 23 participants with prior/current syphilis, 2 (8.7%) had recent infection, 2 (8.7%) reinfection/reactivation, 6 (26%) late latent syphilis, and 13 (57%) serofast status. Prior/current syphilis was associated with lower education (26% of those with syphilis had less than a high school education vs 19% of those without syphilis), greater unemployment (70% with syphilis vs 47% without, *P* = .055), history of incarceration (57% with syphilis vs 25% without, *P* = .002), history of exchanging sex for drugs/money/shelter (22% with syphilis vs 8.5% without, *P* = .046), and number of sexual partners (median, 15 vs 10; *P* = .095). Pregnancy and HIV status were not associated with prior/current syphilis. Two pregnant women screened positive for syphilis on nontreponemal screening tests. One had a recent syphilis infection, with an elevated RPR titer (1:32) and a reactive confirmatory treponemal test. The participant delivered a live birth without adverse obstetrical or infant outcomes. The second pregnant participant had a reactive RPR screening but a nonreactive confirmatory treponemal test and was classified as having a false-positive syphilis test result.

## DISCUSSION

This study suggests that reproductive-age women in the US South face a notable syphilis burden, in particular those women who are otherwise marginalized for reasons of low income, low educational attainment, or prior incarceration. The factors associated with syphilis suggest that the mechanisms underlying syphilis acquisition are multifactorial and that social drivers of health play an important role with shared behavioral factors, structural risk factors, and socioeconomic instability resulting in poor health outcomes [9–11].

STAR is one of the earliest large, longitudinal cohort studies to report syphilis prevalence among reproductive-aged WWH and WWOH living in the US South. This report focuses on baseline syphilis prevalence. Ongoing follow-up within STAR will allow for future analyses of new syphilis diagnoses and longitudinal predictors of infection.

### Limitations

This study has several limitations. The sample size is small; thus, generalizability may be limited. In addition, the sample size did not allow for subgroup analysis specific to recent or recurrent infections. This study focused on serologic diagnosis but lacked specific tests to identify primary or secondary stages of syphilis, as physical examination was not performed, or to differentiate between prior and current infection; prior RPR was also not available. Furthermore, given the few pregnant women with syphilis in this study, we were unable to fully assess complications related to syphilis infection during pregnancy. Additionally, syphilis screening results and data on timing of prior pregnancies relative to syphilis diagnosis were unavailable. This limited assessment of pregnancy-related syphilis risk. Due to the lack of access to medical records to verify prior syphilis diagnoses or treatment history, syphilis history results may be subjected to recall bias as some of the data were self-reported. Additionally, without documented RPR titers, reinfection could not be definitively distinguished from previously treated infection, leading to possible misclassification of the infection stage. As STAR is a longitudinal cohort, this report includes only baseline data. Therefore, new diagnoses of syphilis during follow-up and time-dependent predictors of syphilis acquisition are not included in this analysis.

### Future Research and Application of Findings

Future studies should prioritize longitudinal data collection to track syphilis and HIV coinfection. Since syphilis surveillance focused on primary and secondary infections, which primarily affect men, it is crucial to focus on the female population for screening and intervention.^3^ This study did not formally attempt to estimate causal effects. A thorough causal study incorporating a syndemic approach is necessary to determine if education or other social determinants of health have a direct connection to syphilis risk levels or whether a combination of structural inequalities leads to negative sexual health outcomes [12].

Future health initiatives should implement programs that address patient-level barriers and provider-level gaps, including education-based interventions. The implementation of evidence-based sexual health curricula, adult literacy programs, and peer-led outreach could increase awareness and decrease risk behaviors. Additionally, developing interventions and public policies that can target key social determinants of health will likely aid in decreasing syphilis rates.

## CONCLUSION

Syphilis remains widespread in the southern United States among women. Public health strategies that integrate multifaceted approaches will lead to substantial progress in controlling and reducing syphilis rates and poor reproductive and sexual health outcomes.
